# Isolated Lenticular Coloboma in an Atypical Location With Early Cataract Formation: A Case Report

**DOI:** 10.7759/cureus.102313

**Published:** 2026-01-26

**Authors:** Shahmeer H Noori, Mikes Nomikarios, Evgenia Anikina

**Affiliations:** 1 Medical Education, Hillingdon Hospital, Uxbridge, GBR; 2 Ophthalmology, The Newcastle Upon Tyne Hospitals NHS Foundation Trust, Newcastle, GBR; 3 Ophthalmology, Royal Berkshire NHS Foundation Trust, Reading, GBR

**Keywords:** atypical coloboma, congenital lens anomaly, early cataract, lenticular coloboma, ocular trauma, zonular absence

## Abstract

A coloboma refers to an embryological defect of the eye due to incomplete closure of the embryological fissure in development. Lenticular colobomas are defects of the capsular bag due to an absence of zonules, resulting in a characteristic notching of the lens; however, they are not true colobomas as there is no loss in lens tissue. These defects are often congenital and are typically seen in a unilateral and inferonasal position.

In this case report, we outline the presentation of a 38-year-old female patient who had been experiencing reduced vision and floaters in one eye following an injury. When seen in eye casualty, the patient was noted to be amblyopic with a superotemporal lens coloboma observed in the left eye (LE). The affected LE had a reduced visual acuity of 6/30 compared to 6/6 in the right eye with mild nuclear sclerosis in the affected lens. Following 12 months of conservative management, the lens remained stable with no phacodonesis.

The history of recent trauma and early cataract formation make this case unique. The history of trauma and subsequent visual deterioration raise the possibility of an underlying zonular defect unmasked by injury, highlighting the diagnostic uncertainty that can arise in atypical presentations.

## Introduction

A coloboma refers to a defect formed in the eye from failure of the embryological fissure to close in development, commonly affecting the iris, choroid, retina, or optic nerve [1]. Lenticular colobomas are congenital defects of the capsular bag; however, they are not true colobomas, as they result from an absence of zonules within a quadrant of the eye as opposed to a loss of lens tissue itself [2-4]. This loss or maldevelopment of zonules in the affected region can result in a secondary deformation of the lens due to a lack of tension on the capsule, which can cause flattening or notching of the lens [3,4]. The location and size of lens coloboma vary, most typically being described as unilateral and single in the inferior or inferonasal positions, corresponding with the alignment of the embryological fissure; however, they can occasionally be seen in other positions and associated with other ocular or systemic abnormalities [4-6].

Lens and zonule defects have been reported in the literature to be acquired via alternative non-congenital origin such as through trauma, which is an important distinction to make. However, traumatic zonular defects differ in presentation from lenticular colobomas as they can initially be difficult to detect visually, but can present with lens subluxation, vitreous presentation, or phacodonesis as opposed to the stable equatorial contour seen in congenital lenticular coloboma. In some cases, trauma may unmask a pre-existing zonular abnormality, creating diagnostic uncertainty between the congenital and acquired causes of lens deformation [7,8].

Clinically, lenticular colobomas have been seen to predispose patients to earlier or asymmetric cataract formation for age. On top of this, the loss of zonules seen can increase the risk of lens instability with potential for lens subluxation. These are both important factors to consider when planning for cataract surgery in such patients [3,9,10].

In this case report, we describe the presentation of an atypical isolated supratemporal lenticular coloboma with early cataract formation in the affected lens after a traumatic ocular injury. This case highlights the diagnostic challenge of a congenital lenticular coloboma from acquired zonular pathology in the setting of ocular trauma.

## Case presentation

A 38-year-old woman who was fit and well with no previous ophthalmic history presented to our department after being referred by her optometrist. She reported reduced vision and intermittent floaters in her left eye (LE) for approximately one year following blunt ocular trauma, having sustained a direct blow to her eye by the corner of a mobile phone accidentally swung by her child. She recalled a transient reduction in vision and flashing lights immediately following the injury, for which she saw her optometrist, who reported a stable retina with no tears or detachments. The symptoms resolved after several days, and she did not seek out any further ophthalmic involvement.

The patient described a history of weaker vision in the affected eye since childhood, in the context of significant anisometropia, and presumed amblyopia despite no formal ophthalmological review. She noted a subjective deterioration in vision following the traumatic event.

Approximately one year after the injury, whilst abroad, the patient underwent an ophthalmic assessment and was advised that there were concerns regarding a possible retinal detachment and cataract formation, with urgent surgery recommended. She elected to return to the UK for further evaluation and arranged for review by her usual optometrist. Visual acuity (VA) at that assessment was −0.75/−2.00 ×175 in the right eye (RE) with best-corrected visual acuity (BCVA) of 6/5−1, and −3.50/−3.00 ×140 in the LE with BCVA of 6/9. The optometrist raised concerns over possible retinal pathology in the temporal region of the LE, prompting urgent referral to ophthalmology.

**Figure 1:**
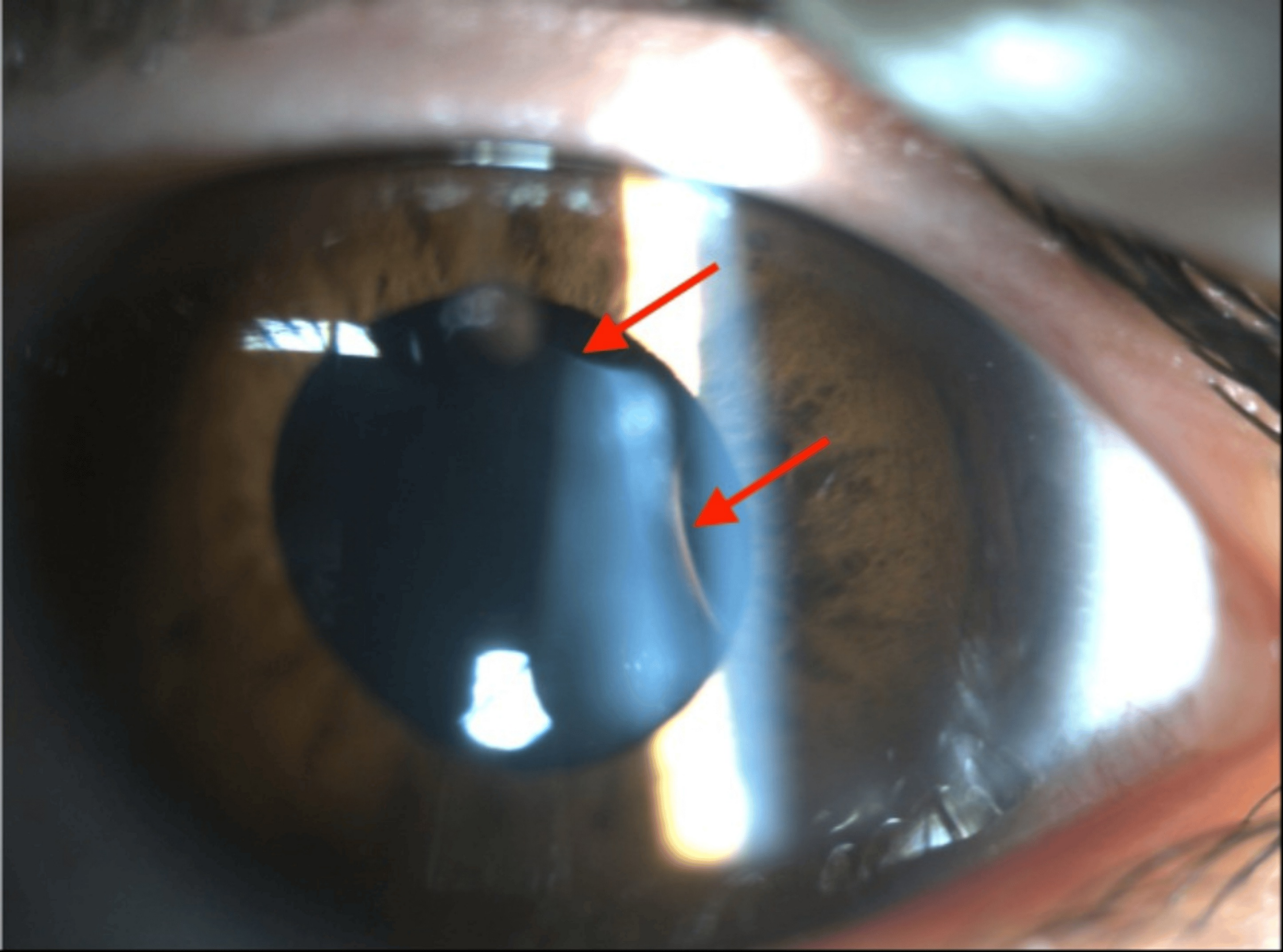
Slit Lamp Photograph of the Left Eye Demonstrating the Lenticular Coloboma Slit-lamp image of the affected eye demonstrating an isolated lenticular coloboma resulting in two discrete notches of the lens equator at 1 o'clock and 3 o'clock positions (arrows), consistent with the absence of zonular support in the involved regions. Early cataractous changes are visible within the lens. The coloboma is located in an atypical position, with no associated iris or chorioretinal coloboma identified on examination.

On examination in the ophthalmic emergency department, her VA was seen to be reduced in the LE (6/6 RE, 6/30 LE) with reduced pupil dilatation in the LE. Slit-lamp examination revealed a lenticular coloboma in the superotemporal quadrant of the LE, with two discrete equatorial notches located at approximately the 1 o’clock and 3 o’clock positions (Figure 1). Dilated fundus examination demonstrated a flat retina with a normal macula. Mild nuclear sclerosis was present in the affected lens. There was no phacodonesis. The remainder of the anterior segment, iris, and fundus examination was unremarkable. B-scan ultrasonography did not reveal any additional abnormalities; however, dedicated anterior segment imaging like ultrasound biomicroscopy (UBM) or anterior segment optical coherence tomography (OCT) was not available; therefore, detailed assessment of zonular integrity was limited. Examination of the RE was normal.

Following the initial assessment, the case was referred to the vitreoretinal team, who reviewed and discussed management options with the patient. Given the lack of capsular support in the LE, surgical intervention would likely require a complex procedure involving lensectomy and scleral-fixated intraocular lens implantation, with a potentially unpredictable visual outcome. In view of the lens being centrally positioned and relatively stable, a conservative watch-and-wait approach was agreed, with monitoring for progression of nuclear sclerosis or lens displacement. The patient was followed up in the clinic for one year after the initial presentation, during which time the lens remained stable with no evidence of progressive subluxation. Her VA in the LE fluctuated during follow-up and was recorded as 6/12 at the last follow-up appointment.

## Discussion

Lenticular colobomas are rare anomalies of the lens characterised by localised absence or weakening of the zonular apparatus, often resulting in a notch-like defect in the lens equator [1,2]. Unlike typical colobomas involving the iris, choroid, or retina that follow the embryonic fissure line, lenticular colobomas are not true colobomas in the embryological sense but represent secondary deformations due to zonular deficiency or developmental disruption [1-4].

In this case, our patient presented with an atypical lenticular coloboma in the superotemporal quadrant of the LE, with two distinct lenticular defects, a rare and unusual finding. While congenital lenticular colobomas are typically located inferonasally in association with colobomas of other ocular structures, the location and multiplicity of defects in this case are atypical and raise important considerations about pathogenesis [5,6]. To our knowledge, only one prior case of a superotemporal lenticular coloboma has been identified from the literature in a presumedly asymptomatic adult patient [11]. What makes our case unique is the possible role of trauma in the patient’s presentation, raising an important discussion on the pathogenesis of the defect seen, which has not been previously described in association with a lenticular coloboma.

The patient’s history of ocular trauma prior to the onset of symptoms could suggest a possible trauma-induced zonular disruption or unmasking of a pre-existing zonular abnormality. Despite the lack of documented ophthalmic history prior to the traumatic episode, the presence of a longstanding weakness in vision of the LE since childhood in the context of significant anisometropia supports an underlying congenital component. This temporal association between the visual decline and trauma therefore suggests a contributory role for trauma in this case rather than supporting the evidence for an acquired lenticular coloboma.

Interestingly, there was no phacodonesis, and the lens remained stable over a year of follow-up. This absence of generalised lens instability supports the likelihood of a localised rather than diffuse zonular compromise. But it is important to note that this zonular loss was inferred from slit-lamp examination and characteristic lens morphology, as dedicated anterior segment imaging such as UBM or anterior segment OCT was not available. The coexisting mild nuclear sclerosis may have contributed to visual impairment but is unlikely to be the sole cause, given the focal structural abnormality observed.

This case adds to the limited literature on atypical lenticular colobomas, particularly those occurring in non-inferonasal locations and possibly acquired later in life. Prior reports have emphasised congenital associations, systemic syndromes, and ocular colobomas along the embryonic fissure. However, trauma-related lenticular deformities resembling colobomas, though rarely documented, must be considered, especially in isolated, quadrant-specific presentations. Although very rare, acquired lens colobomas secondary to ocular surgeries are described in the literature [12].

From a management perspective, conservative management was deemed appropriate given the stability of the lens and absence of progressive subluxation or visually significant cataract. Surgical intervention, such as lens extraction with or without capsular support, remains reserved for cases with progressive visual decline, lens instability, or coexistent pathology. The fluctuation in VA over time underlines the importance of individualised monitoring in such cases.

This case also highlights the importance of detailed anterior segment evaluation, use of imaging modalities like B-scan ultrasonography, and a thorough history in diagnosing atypical lenticular anomalies. It also raises awareness about trauma as potential aetiology in the differential diagnosis of localised lens deformities.

This case has several important limitations. The absence of prior ophthalmic records or historical documentation of lens status limits definitive classification of the defect as congenital or acquired. In addition, the retrospective nature of the trauma history restricts detailed assessment of the mechanism of injury and its potential contribution to the observed findings. Finally, advanced anterior segment imaging, such as UBM or anterior segment OCT, was not available and may have provided further insight into zonular integrity.

## Conclusions

This case describes a rare presentation of a superotemporal lenticular coloboma in an adult patient, with clinical features suggesting a possible trauma-related or trauma-unmasked aetiology. While lenticular colobomas are classically described as congenital and inferonasal, this case highlights that atypical locations and adult presentations can occur and may be associated with preceding ocular trauma. The observed stability of the lens over the 12-month follow-up period, with the absence of further subluxation or phacodonesis, supports the initial conservative management approach in selected patients. Nevertheless, recognition of the zonular weakness seen is clinically relevant, particularly when considering future cataract surgery, given its influence on intraoperative risk, which, thus, may influence surgical planning.

Although a definitive causal relationship between trauma and lenticular coloboma formation cannot be established in this case, the temporal association and clinical findings suggest that trauma may play a role in unmasking pre-existing zonular weakness or contributing to acquired lens deformity. This reinforces the importance of thorough history taking, detailed anterior segment examination and appropriate imaging in patients presenting with localised lenticular abnormalities. Further case reports and larger case series are needed to better demonstrate the pathogenesis and clinical implications of atypical and potentially acquired lenticular colobomas and to clarify the role of trauma in their development.
